# Occurrence and characterization of methicillin-resistant staphylococci from bovine mastitis milk samples in Finland

**DOI:** 10.1186/1751-0147-55-61

**Published:** 2013-08-28

**Authors:** Veera Gindonis, Suvi Taponen, Anna-Liisa Myllyniemi, Satu Pyörälä, Suvi Nykäsenoja, Saara Salmenlinna, Laura Lindholm, Merja Rantala

**Affiliations:** 1 Faculty of Veterinary Medicine, Department of Production Animal Medicine, University of Helsinki, Koetilantie 7, 00014 Helsinki, Finland; 2 Research and Laboratory Department, Food and Feed Microbiology Research Unit, Finnish Food Safety Authority Evira, Mustialankatu 3, 00790 Helsinki, Finland; 3 Faculty of Veterinary Medicine, Department of Production Animal Medicine, University of Helsinki, Paroninkuja 20, 04920 Saarentaus, Finland; 4 Department of Infectious Disease Surveillance and Control, National Institute for Health and Welfare, PO Box 30, 00271 Helsinki, Finland; 5 Department of Infectious Disease Surveillance and Control, National Institute for Health and Welfare, PO Box 57, 20521 Turku, Finland; 6 Faculty of Veterinary Medicine, Department of Equine and Small Animal Sciences, University of Helsinki, Viikintie 49, 00014 Helsinki, Finland

**Keywords:** Staphylococci, Methicillin resistance, SCC*mec*, Bovine, Mastitis, *mec*A, *mec*C

## Abstract

**Background:**

Methicillin-resistant staphylococci (MRS) are increasingly being isolated in bovine mastitis. The aim of our study was to evaluate the occurrence of MRS in Finnish mastitis milk samples and characterize the MRS isolates using molecular methods.

**Results:**

Methicillin-resistant *S. aureus* (MRSA) was a rare finding in bovine mastitis in Finland. Only two out of 135 (1.5%) *S. aureus* isolates were positive for *mec* genes. One of these carried *mec*A and was of *spa* type t172, SCC*mec* type IV and ST375, and the other harboured *mec*C, being *spa* type t3256, and ST130. MRSA ST375 is common among human MRSA isolates in Finland, but this is the first report in the country of bovine *mec*C MRSA. In coagulase-negative staphylococci (CoNS) originating from bovine mastitis, methicillin resistance was more common. In the two CoNS collections studied, 5.2% (17/324) and 1.8% (2/110) of the isolates were *mecA* positive. Eighteen of these were methicillin-resistant *S. epidermidis* (MRSE), which were divided into 6 separate PFGE clusters. One pulsotype was detected in different parts of the country, indicating clonal spread. Most MRSE (13/18) were of SCC*mec* type IV, one was of type V and four were non-typeable. Comparison with a human staphylococcal database indicated that bovine MRSE strains were not closely related to human MRSE isolates.

**Conclusions:**

The occurrence of MRS, especially MRSA, in bovine mastitis in Finland was low. Most methicillin-resistant bovine CoNS are MRSE, and we found evidence of a bovine MRSE strain that may spread clonally. This is the first report of a Finnish bovine isolate of MRSA_*mecC*_ ST130. The study provides a baseline for further MRS monitoring.

## Background

Staphylococci are common etiological agents of bovine mastitis. Infections caused by methicillin-resistant staphylococci (MRS) are more difficult to treat and may pose a public health risk [1]. Methicillin-resistant *Staphylococcus aureus* (MRSA) was first reported in cows in 1972, when Devriese and co-workers found 5.2% of Belgian dairy farms MRSA positive [2]. Thirty years later, MRSA became a sporadically reported finding from bovine mastitis [3]. MRSA mainly spreads clonally. Some MRSA isolates from bovine mastitis are thought to be bovine and some of human origin [4,5]. Clonal transmission between farmers and dairy cows has been shown to occur [1]. More recently, livestock-associated MRSA (LA-MRSA) strains of MRSA ST398 and strains carrying MRSA_*mecC*_ have emerged in humans [5-7]. These have also been isolated in bovine mastitis [5,8]. MRSA ST398 has been suggested to have developed in humans as a methicillin-sensitive *S. aureus* (MSSA) strain that was transferred to livestock, first to pigs and then to cattle, and acquired the *mecA* gene [9]. MRSA_*mecC*_, however, was first found in both human and bovine samples in 2011, and several different sequence type lineages of MRSA_*mecC*_ have been recognized [5,10].

Methicillin-resistant coagulase-negative staphylococci (MR-CoNS) have also been isolated in bovine mastitis [11-14]. They may be either species such as *S. epidermidis* or *S. haemolyticus*, which are commonly encountered in both human and animal specimens, or species that are mainly associated with bovine mastitis, such as *S. simulans* or *S. chromogenes*[12,13]. In particular, methicillin-resistant *S. epidermidis* (MRSE) has been a common finding: two recent studies from the Netherlands and the USA reported that over 30% of *S. epidermidis* isolated in bovine mastitis were MRSE [13,14]. In a German study, eight out of 14 *S. epidermidis* were MRSE [12]. There is evidence of clonal transmission of MR-CoNS between humans and dairy cows [15], and MRSE has been thought to spread clonally within a herd of dairy cows [14].

Methicillin resistance in staphylococci is due to penicillin-binding protein 2α (PBP2α), which is encoded by *mec* genes. The *mec* genes are embedded in large mobile elements called SCC*mec* (staphylococcal cassette chromosome *mec*) [16], which also carry a cassette chromosome recombinase (*ccr*) gene complex and J regions (joining regions J1–J3) [17]. To date, 11 different classes (I–XI) of SCC*mec* in MRSA have been reported, and these are further divided into subtypes according to variations in the J regions [18]. At least classes I–V and XI of MRSA, and classes III–V of MR-CoNS, along with several variants, have been isolated in bovine mastitis [4,5,12,19-21]. MR-CoNS generally carry a vast variety of SCC*mec* elements and are currently considered to be the likely reservoir of the different types of *mecA* gene in MRSA [22].

The aim of this study was to 1) estimate the occurrence of MRS in bovine mastitis in Finland, 2) characterize the MRS isolates collected from bovine mastitis, and 3) compare them with available human isolates.

## Methods

### Bacterial isolates

To investigate the occurrence of methicillin resistance in staphylococcal species isolated from bovine mastitis, three sets of bacterial isolates were included in the study: one collection of *S. aureus* (collection 1) and two collections of CoNS (collections 2 and 3) (Table 1). From each collection, one isolate per staphylococcal species per cow was included in the present study. In rare cases where two or more isolates of the same species of the same cow were available, the most resistant isolate was chosen. All isolates were confirmed to be staphylococci by their typical colonial appearance, a positive catalase test and staining as Gram-positive cocci in clusters. Preliminary differentiation between *S. aureus* and CoNS was performed using a tube coagulase test (BD BBL Rabbit Coagulase Plasma, Becton Dickinson, NJ, USA).

**Table 1:** Collections of staphylococci isolated from bovine mastitis included in the study*

**Material**	**Origin**	**Year**	**Isolates**	**Farms**	**OxaMIC = R**	**MRS isolates**	**MRS-positive farms**	**MRS species**
			**n**	**n**	n (%)***	**n (%)**	**n (%)**	
1) *S. aureus* isolates from the Finnish Food Safety Authority (Evira)	Subclinical and clinical mastitis cases nationwide.	2003–2008	135	117	18 (13.3)	2 (1.5)	2 (1.7)	MRSA
2) CoNS isolates from Bovine Mastitis Survey material (Pitkälä et al., 2004)	Subclinical and clinical mastitis, randomized sample of a nationwide bovine mastitis survey.	2001	324	179	34 (10.5)	17 (5.2)	16 (8.9)	All MRSE, 2 isolates originating from the same farm
3) CoNS isolates from the University Ambulatory Clinic** material (Taponen et al., 2006)	Subclinical and clinical mastitis, on-call area of the University Ambulatory clinic.	1999–2002	110	57	6 (5.5)	2 (1.8)	2 (3.5)	1 MRSE 1 MR-*S. fleurettii*

* All isolates have been selected from the collections so that there is only one isolate per staphylococcal species per cow.

** The Ambulatory Clinic of the Department of Production Animal Medicine, Faculty of Veterinary Medicine, University of Helsinki.

*** The resistance breakpoints used for oxacillin were >2 μg/ml for bacterial collections 1 and 2, and >1 μg/ml for collection 3 (see text for details).

### Antimicrobial susceptibility testing

Susceptibilities of the isolates were tested for penicillin, oxacillin, tetracycline, erythromycin, clindamycin, trimethoprim, chloramphenicol, vancomycin, gentamicin and ciprofloxacin by using a broth microdilution method (VetMIC™, SVA, Sweden) according to the existing standards [23,24]. Oxacillin results for bacterial collection 2 were interpreted using a resistance breakpoint of >2 μg/ml (Table 2) [25]. EUCAST epidemiological breakpoints (http://www.eucast.org) were applied to all other susceptibility test results. Beta-lactamase production by MRS isolates was tested with Cefinase disks (BD BBL Paper Disk for the Detection of β-Lactamase Enzymes, Becton-Dickinson, NJ, USA).

**Table 2:** **Characteristics of the methicillin-resistant *S. epidermidis *(MRSE) from bovine mastitis of the study (n = 18)**

**PFGE Cluster**	**Isolate no.**	Oxacillin MIC (μg/ml)*	Antimicrobial resistance profile*	**β-lactamase disk test**	**BlaZ gene**	**SCC*mec *type (*ccr *type, *mec *class)**
A	658	>4	PEN, OXA	+	+	IV (2B)
B	1314	>4	PEN, OXA	-	+	NT (2 -)
C_1_	1018	>4	PEN, OXA, TET, ERY	+	+	IV (2B)
C_1_	1289	4	PEN, OXA, TET	+	+	IV (2B)
C_1_	1439	4	PEN, OXA, TET, CHL	+	+	IV (2B)
C_1_	1834	>4	PEN, OXA, TET	+	+	IV (2B)
C_1_	2156	>4	PEN, OXA, TET	+	+	IV (2B)
C_1_	28 VT	>4	PEN, OXA, TET	+	+	IV (2B)
C_2_	1532	>4	PEN, OXA, TET	+	+	IV (2B)
C_3_	1391**	4	PEN, OXA, TET	+	+	IV (2B)
C_3_	1409**	4	PEN, OXA, TET	+	+	IV (2B)
C_4_	775	>4	PEN, OXA, TET	+	+	V (5C2)
D_1_	641	4	PEN, OXA, TET	+	+	IV (2B)
D_1_	1750	>4	PEN, OXA, TET	-	-	IV (2B)
D_2_	990	>4	PEN, OXA, TET	+	+	NT (2,5C2)
E_1_	9	2	PEN, OXA, TET, ERY	+	+	NT (2,5B)
E_2_	1222	>4	PEN, OXA, TET, ERY	+	+	NT (− −)
F	922	>4	PEN, OXA	+	+	IV (2B)

Penicillin PEN, oxacillin OXA, tetracycline/oxytetracycline TET, erythromycin ERY, chloramphenicol CHL.

* Based on the results from Pitkälä et al. 2004 (Survey material), except for isolate 28 VT, which is from the Ambulatory Clinic material.

** Isolates originated from the same farm.

*NT* Non-typeable using the method described.

### Extraction of DNA

Chromosomal DNA for ribotyping and PCR was prepared either using a previously described method [26] or with the MasterPure Gram Positive DNA Purification Kit (Epicentre Biotechnologies, Illumina Inc., Madison, WI, USA), High Pure PCR Template Preparation Kit (Roche, Basel, Switzerland) or Easy-DNA Kit (Invitrogen, Life Technologies, CA, USA).

### Bacterial identification by *nuc*-PCR and ribotyping

Identification of MRSA isolates to the species level was verified by PCR using primers specific for *S. aureus* thermostable nuclease, the *nuc* gene [27]. MR-CoNS isolates were analysed with the 16S and 23S rRNA gene restriction fragment length polymorphism method (ribotyping), as described previously [28], and identified by comparison in a numerical similarity analysis of the ribotype patterns with a ribotype library using the BioNumerics 4.6 software package (Applied Maths, Kortrijk, Belgium).

### Detection of *mec*A, *mec*C, *bla*Z and PVL genes by PCR

Staphylococci were tested for carriage of the *mec*A gene by *mec*A PCR [29] with MRSA ATCC 29213 as a positive control. The selection criteria for *mec*A screening were set to include all isolates exceeding the oxacillin resistance breakpoint, and in addition, to include a representative number other isolates, especially those having an oxacillin MIC near the breakpoint. As a result, we included 63 *S. aureus* isolates (collection 1): all isolates having oxacillin MIC ≥ 4 μg/ml (n = 18); 80% of isolates having an oxacillin MIC of 2 μg/ml (n = 36); and 9 isolates with low oxacillin MICs (≤ 1 μg/ml). According to oxacillin histograms of the CoNS collections, separation into susceptible and resistant populations was not clearly evident. Therefore, from collection 2 we screened 238 CoNS isolates as follows: all isolates with MIC ≥ 4 μg/ml (n = 34), as well as all isolates with MIC = 1–2 μg/ml (n = 200) and 4 isolates with lower MICs. Collection 3 was smaller and all CoNS isolates were therefore included for *mec*A PCR (n = 110).

All *mec*A-negative *S. aureus* and *mec*A-negative CoNS isolates with an oxacillin MIC of >2 μg/ml (n = 42) were tested for the presence of the *mec*C gene by using previously described primers for a 138 bp PCR product [10]. MRSA_LGA251_ was used as a positive control. The *mec*C PCR reaction mixture contained 0.2 μM of both primers, 200 μM of each deoxynucleoside triphosphate, 1× buffer, 1U of polymerase (DyNAzyme DNA Polymerase Kit, Finnzymes, Espoo, Finland), and 1 μl of DNA template in a final volume of 50 μl. The PCR cycling conditions were as follows: initial denaturation at 94°C for 5 min; 35 cycles of denaturation at 94°C for 30 s, annealing at 58°C for 1 min, and elongation at 72°C for 1 min; and final elongation at 72°C for 10 min. Positive *mec*C PCR results were confirmed by sequencing of the PCR product at the Institute of Biotechnology, University of Helsinki. The sequence data were analysed with CLC Main Workbench software (version 6.6.2, CLCbio, Aarhus, Denmark). All *mec*A- or *mec*C-positive isolates were considered truly methicillin resistant (MRSA or MR-CoNS) and were further tested for the *bla*Z gene by a previously described method [30]. *S. aureus* ATCC 29213 was used as a positive control and *S. aureus* ATCC 25923 as a negative control [31]. In addition, MRSA isolates were tested for the carriage of genes for Panton-Valentine leucocidin (PVL), *lukS-PV* and *lukF-PV*[32].

### SCC*mec* typing

The SCC*mec* types of isolates were assigned according to two previously described multiplex PCRs, M-PCR1 and M-PCR2 [33], with slight modifications. M-PCR1 and M-PCR2 were carried out using 2U of DyNAzyme I and 2U of DyNAzyme EXT DNA polymerases (Finnzymes, Espoo, Finland), respectively. The amount of DNA template was 200 ng in a final volume of 25 μl in both multiplex PCRs. The following strains of *S. aureus* were used as positive controls: NCTC 10442 (ccr1 and mec product B), ATCC BAA-1720 (ccr2, mec product A), ATCC BAA-43 (ccr3), ATCC BAA-42 (ccr4) and JCSC 6944 (ccr5, mec product C2).

### *Spa* sequence typing and multilocus sequence typing (MLST) of MRSA

The region encoding the *Staphylococcus* Protein A (*spa*) was amplified as previously described [34]. The gene product was sequenced and the repeat region was analysed using BioNumerics (version 6.5, Applied Maths, Belgium). MLST was performed as previously described [35]. Sequencing results were compared against the MLST database and the sequence types were determined ( http://www.mlst.net).

### Pulsed-field gel electrophoresis

MRSE isolates were genotyped by PFGE using a previously described method [36] with slight modifications: DNA fragments were separated on 1% agarose made in 100 ml 0.5× TBE (casting frame 14 by 13 cm) by using a Chef DR III system (Bio-Rad, USA) with 2000 ml of 0.5× TBE as the running buffer. PFGE patterns were analysed using Bionumerics software (version 5.10, Applied Maths, Belgium). For interpretation, a similarity cut-off of 80% and a difference of ≤6 in PFGE patterns were used in assigning the isolates into clusters; identical patterns were considered as the same pulsotype, and a difference of 3–6 bands as subtypes of the same cluster [37]. The PFGE patterns of the bovine MRS isolates were compared with the human staphylococcal culture collection of the National Institute of Health and Welfare (THL).

## Results

Two out of 135 *S. aureus* isolates originating from bovine mastitis (1.5%) were MRSA, one isolated in 2005 and the other in 2006 from different herds. Both MRSA strains had oxacillin MIC of >16 μg/ml. The MRSA strain isolated in 2005 was of *spa* type t172, SCC*mec* type IV, ST 375, which corresponds to the PFGE type FIN-4 [37]. It carried the *bla*Z gene and was PVL negative. In addition to oxacillin the strain was phenotypically resistant to penicillin and trimethoprim, but susceptible to other tested antimicrobials. The MRSA strain isolated in 2006 carried *mec*C instead of *mec*A, but was negative in *bla*Z PCR, although phenotypically it produced beta-lactamase. This strain was highly susceptible to all tested antimicrobials except penicillin and oxacillin. Sequencing verified the *mec*C PCR product to be identical to the *mec*C of the MRSA LGA251 [5]. The strain was of the ST130 and *spa* type t3256, and was PVL negative. SCC*mec* of this strain was not recognised by the used method.

**Figure 1:**
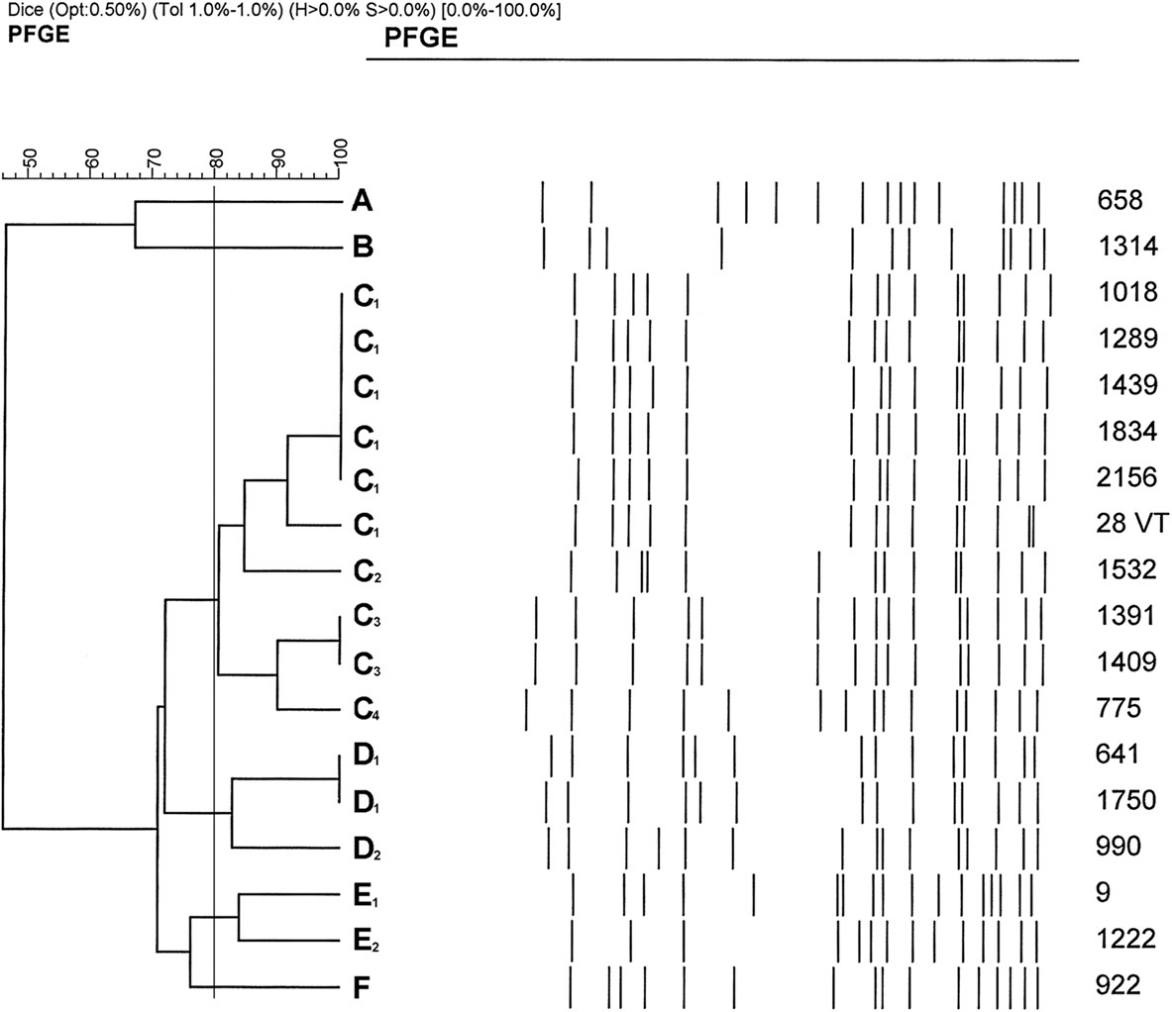
PFGE patterns of the MRSE strains isolated from bovine mastitis in Finland.

Seventeen out of 324 (5.2%) CoNS isolates from the national mastitis survey (bacterial collection 2) were positive for *mec*A. All of them were ribotyped as *S. epidermidis*. In this collection, 21.5% of all *S. epidermidis* isolates (17/79) were MRSE. Based on the PFGE pattern analysis, the strains of MRSE could be divided into three clusters of 2, 10 and 3 isolates, and 3 singletons (Figure 1). Twelve isolates were of SCC*mec* type IV and one isolate was assigned to type V. Four isolates were non-typable: two of them carried both *ccr* products 2 and 5, and the two other did not carry any *mec* products recognized by the methods used. Five of the cluster 2 isolates were identical in PFGE (Figure 1), and they all possessed the type IV SCC*mec* cassette. When compared with human MRSE and MSSE (methicillin sensitive *S. epidermidis*) strains, no similarity over 83.9% was detected. In the samples collected at the Ambulatory Clinic (bacterial collection 3), 2 out of 110 (1.8%) CoNS were resistant to methicillin. The isolates were identified as *S. epidermidis* and *S. fleurettii* by ribotyping. Altogether, nine *S. epidermidis* isolates were recognised in collection 3. MRSE (isolate 28 VT in Figure 1 and Table 2) was of SCC*mec* type IV and belonged to cluster C. *S. fleurettii* was non-typeable by the SCC*mec* protocol used.

No resistance to more than two classes of antimicrobials (multiresistance) was detected in the bovine MRSA, but four out of 18 MRSE of the study were multiresistant (Table 2).

## Discussion

The occurrence of MRSA in the present study was low, totalling 1.6%. However, 5.2% and 1.8% of the investigated CoNS isolates were *mecA* positive in bacterial collections 2 and 3, respectively. On the farm level, MRS occurred on 8.9% and 3.5% of the farms. These are expected results, as MR-CoNS are more common than MRSA [38]. In earlier studies, the occurrence of MRSA (percentage of *mecA*-positive isolates out of all isolates in the given *S. aureus* collection) from bovine mastitis milk from Japan, Switzerland, Korea and Belgium has been 1.1%, 1.4%, 1.6% and 9.3%, respectively [8,11,20,39]. The occurrence of MR-CoNS in previous studies has been between 1.6 and 13.5% [11-14]. However, due to methodological differences, comparison between the studies is difficult.

The MRSA ST375 found in 2005 was of a type that is frequently isolated in human specimens in Finland [40], and it is likely to be of human origin. Human infections due to MRSA ST375 are usually community acquired and isolates of this clone are typically very susceptible to other antimicrobials except betalactams. This was also the case in our study. A follow-up study was performed in 2008 in the same farm where ST375 was first detected in 2005 (data not shown). Milk specimens as well as environmental specimens were collected. An identical MRSA ST375 was again detected in one of the milk specimens, but in addition, another MRSA strain was discovered from a separate milk specimen. The latter strain had the same *spa* type t172 as the previously identified MRSA ST375, but showed changes in the PFGE pattern and was thus classified as a subtype of FIN-4 by PFGE. Because in 2008 the cows on this farm were different from those sampled in 2005, the ST375 strain detected in the cows could have originated from the herd staff carrying the strain that evolved in them, and might have been re-transmitted to the susceptible cows over the years. Sampling of humans may have given additional information to the epidemiology of MRSA on the farm, but was not performed, as the people on the farm rejected further evaluation.

The MRSA_*mecC*_ t3256 ST130 found in the present study is of the same *spa* type as two other *mecC*-containing MRSA isolates from Denmark [10]. Our strain was very susceptible to other antimicrobials, which has been also a common finding in earlier reports. *MecC*-carrying MRSA strains have also previously been encountered in specimens from both humans and dairy cows in UK, Ireland and France [5,7,10,41]. A recent study revealed that *mec*C carrying *S. aureus* strains belong usually to two distinct clonal lineages, CC 130 and CC 2361, but there are several distinct *spa* types [42]. Moreover, the results of the same study showed that human infections associated with *mec*C MRSA are usually community acquired and often detected in humans living in rural area. There is also strong evidence that *mec*C MRSA is transmitted between ruminants and humans [42,43]. The role of bovines and other ruminants in the epidemiology of these MRSA lineages in humans warrants further investigation. Sporadic cases of human MRSA_*mecC*_ have also been detected in Finland (THL, unpublished data), but molecular typing results were not yet available for comparison.

All but one of 19 MR-CoNS of the present study was MRSE. The occurrence of MRSE among *S. epidermidis* was lower than in previously published studies [12-14]. According to the PFGE results, the MRSE were assigned into six different clusters (Figure 1). Five out of ten cluster C isolates were identical in the PFGE, although they originated from different parts of the country and no common denominator could be seen. It seems possible that this pulsotype spreads clonally. No close match was found for the bovine strains of MRSE in the human staphylococcal database of THL. However, there is no continuous molecular surveillance of human MRSE, and the database only covers genotypes from a limited number of isolates obtained from hospitalized patients. One human MRSE isolate of the THL database had a similarity of 83.9% with the cluster F singleton MRSE 922 of the present study, and would therefore be classified as a subtype of cluster F. *S. epidermidis* is a common cause of hospital-acquired infections in humans, and the hospital-acquired strains are mostly resistant to methicillin [38]. Human isolates are often multiresistant, whereas the strains found in this study were susceptible to most classes of antimicrobials tested. This also suggests that cattle and humans harbour different strains of MRSE.

The SCC*mec* types IV and V found in the MRSE of the present study are considered as community-acquired SCC*mec* types in MRSA. Both types have previously been encountered in bovine MRSE isolates [12,44]. It is also common that MR-CoNS carry SCC*mec* elements that are non-typeable by the established methods [22]. SCC*mec* types of bovine MRSE seem to have variation like the isolates originating from humans [45], and bovine MRSE may spread clonally between dairy farms the same way as strains of MRSA and MRSE spread in humans.

MRSA ST375 and most MRSE in this study (17/18) carried the *blaZ* gene encoding beta-lactamase, causing penicillin resistance. No *blaZ* gene was detected in the MRSA ST130 carrying the *mecC* gene, although it was phenotypically beta-lactamase positive. The most probable reason for a negative result in *bla*Z PCR is that this strain carries a divergent *bla*Z gene as described earlier in *S. aureus* LGA 251 type strain or other strains belonging to *S. aureus* clonal complex CC 130 with SCC*mec* XI [5,7]. These studies reported that in this particular SCC*mec* cassette the nucleotide sequences of the *mec* and genes regulating its function, *mec*I and *mec*R1, as well as sequences of the *bla*Z and *ccr* genes have many differences compared to the respective genes carried by other SCC*mec* types. For instance, similarities of divergent *bla*Z gene sequence of the SCC*mec* XI are only between 44–68% at the protein level when compared to other staphylococcal beta-lactamase genes at the GenBank database (http://www.ncbi.nlm.nih.gov). The *bla*Z gene primers used in this study are not able to detect the *bla*Z gene variant of the SCC*mec* XI.

In many countries, mastitis is treated without bacteriological diagnosis. Currently, almost 80% of milk specimens from bovine mastitis in Finland are diagnosed by a commercial PCR test that identifies the bacteria in the specimen. For resistance, however, it only detects penicillin resistance mediated by the *bla*Z gene. Therefore, it is likely that cases of MRS mastitis mostly go unnoticed. These can, in fact, be incorrectly reported as penicillin susceptible, if staphylococci with divergent *bla*Z variant will emerge and the primers of the test in question are not covering the variant. Incorrect susceptibility information can lead to inappropriate treatment strategy that may, in part, select for already existing strains of MRS. Treatment with cephalosporins but also with other beta-lactams has been shown to select for MRSA in humans [46]. These antibiotics are commonly used in dairy cows. A reliable susceptibility testing method is therefore of the utmost importance. National Finnish recommendations for MRSA of animal origin [47] state that cows with MRSA should primarily be culled, the animal segregated or the infected quarters dried off to stop the MRSA from spreading to other cows and people. Methicillin resistance is still rare in Finnish CoNS of bovine origin, but MRSA-related management recommendations could also be applied to cows carrying MRSE. To detect changes in methicillin resistance in bovine staphylococci, regular national surveillance is needed.

## Conclusions

The occurrence of MRS in dairy cows in Finland was low. The results of this study can be used as a baseline for further surveillance. According to our results, MRSE is the most likely MRS in bovine mastitis, but MRSA is expected to be a sporadic finding. MRSE can spread clonally among the bovine population. Of the two MRSA strains found in this study, MRSA ST375 is also common among human MRSA isolates in Finland. This is the first report of bovine MRSA ST 130 carrying the *mec*C gene in our country.

Staphylococci isolated in bovine mastitis should be routinely tested for methicillin resistance. Surveys to re-assess the situation should be conducted at regular intervals for early intervention and the prevention of epidemic strains from spreading in the population. Maintaining low prevalence of MRS mastitis in Finland may succeed through continuous education of veterinarians and farmers on the importance of reliable susceptibility testing, hygiene and judicious use of antibiotics.

## Competing interests

The authors declare that they have no competing interests.

## Authors’ contributions

VG participated in the planning of the study, PCR, PFGE and ribotyping, and analysed the data and drafted the manuscript. ST participated in the planning of the study, performed ribotyping and participated in writing the manuscript. A-LM and SN provided isolates from the bacterial collection at Evira, carried out antimicrobial susceptibility testing, PCR*,* and SCC*mec* typing, SN also did MLST typing. SP participated in planning and coordination of the study, collection of isolates, and preparing the manuscript. SS participated in MLST typing, and together with LL helped with PFGE analysis and compared the isolates of the study with the respective human MRS strains from the reference laboratory database of THL. MR participated in analysing the data and preparing the manuscript and act as a supervisor of VG. All authors participated in preparing the manuscript, and read and approved the final manuscript.
